# Increasing hypoxia progressively slows early embryonic development in an oviparous reptile, the green turtle, *Chelonia mydas*

**DOI:** 10.1098/rsos.220709

**Published:** 2022-08-31

**Authors:** David M. Adams, Sean A. Williamson, Roger G. Evans, Richard D. Reina

**Affiliations:** ^1^ Monash University, Clayton, Victoria 3800, Australia; ^2^ Cardiovascular Disease Program, Biomedicine Discovery Institute and Department of Physiology, Monash University, Clayton, Victoria 3800, Australia; ^3^ Pre-clinical Critical Care Unit, Florey Institute of Neuroscience and Mental Health, University of Melbourne, Parkville, Victoria 3010, Australia

**Keywords:** embryonic arrest, white spot, oxygen availability

## Abstract

Green turtle (*Chelonia mydas*) embryos are in an arrested state of development when the eggs are laid, but in the presence of oxygen, arrest is broken and development resumes within 12–16 h. However, the precise oxygen level at which embryos break arrest and continue development is not known. To better understand the impact of oxygen concentration on breaking of arrest and early embryonic development, we incubated freshly laid eggs of the green sea turtle for three days at each of six different oxygen concentrations (less than or equal to 1%, 3%, 5%, 7%, 9% and 21%) and monitored the appearance and growth of white spots on the shell, indicative of embryonic development. As reported previously, white spots did not develop on eggs incubated in anoxia (less than or equal to 1% oxygen). For all other treatments, mean time to white spot detection and white spot growth rate varied inversely with oxygen concentration. In nearly all cases the difference between eggs at different oxygen levels was statistically significant (*p* ≤ 0.05). This suggests that sea turtle embryonic development may respond to oxygen in a dose-dependent manner. Our results indicate that the development of green turtle embryos may be slowed if they are exposed to the most hypoxic conditions reported in mature natural nests.

## 1. Introduction

Pre-ovipositional embryonic developmental arrest is a phenomenon found in some oviparous reptiles in which embryonic development is suspended *in utero* and does not resume until after oviposition [1,2]. Early embryonic development and differentiation proceed normally only as far as gastrulation, at which stage the embryo enters a state of developmental arrest [1,3,4]. This unique reproductive strategy is expressed by all turtle species in which it has been investigated [1,5,6], several species of squamate lizards [7,8] and the tuatara [3], and has a substantial influence over life-history traits [9–12] and reproductive success [13–17]. It also represents a physiological barrier to the evolution of viviparity [18,19].

Pre-ovipositional arrest is most likely an adaptive characteristic for at least three reasons. First, it grants females the ability to delay nesting, without risk of embryos developing beyond the point where they may be negatively impacted by extended egg retention [1] when conditions at a chosen nesting site are unsuitable [10,20]. Second, there is evidence that pre-ovipositional arrest provides survival benefits to the offspring due to its synchronizing effect on development and subsequent nest emergence [21,22], although the anti-predation benefits provided by simultaneous emergence may depend on the type of predator [23]. Finally, since the fragile extra-embryonic membranes responsible for gas exchange have not yet fused with the inner shell membrane, arrested embryos are protected from movement-induced mortality when the eggs fall into the egg chamber during oviposition [24,25].

In green sea turtles (*Chelonia mydas*), pre-ovipositional arrest is facilitated and maintained by oviducal hypoxia within gravid females [19] and development resumes (i.e. arrest is ‘broken’) 12 to 16 h after exposure to normoxic conditions [19,26]. Pre-ovipositional arrest in many, if not all, sea turtle species can be artificially extended after oviposition by hypoxic incubation [12,17,19,25], but only if the eggs are placed in hypoxia before the arrest is broken. Otherwise, the embryos perish [26]. The effect of oviducal hypoxia on early embryonic development has been demonstrated [19], but previous studies of pre-ovipositional arrest have only used 1% and 21% oxygen treatments, so the precise concentration of oxygen required for the resumption of embryonic development remains unknown. It may be that there is a critical oxygen level that triggers development and that once this threshold is exceeded, development proceeds at a rate consistent with standard reptile developmental tables that couple growth and temperature. Alternatively, it may be that near-anoxic conditions are required to maintain arrest, but even small amounts of oxygen will permit some level of development to proceed. If the latter could be demonstrated, it would suggest that embryonic development in turtles is a more complex physiological process than previously understood, dependent upon not only temperature and moisture but also variably affected by ambient oxygen concentration. Transcriptional activity of green turtle embryos incubated in hypoxia is substantially different to embryos immediately post-oviposition and when incubated in ambient oxygen [27], although the functional significance of the affected genes remains unknown. Knowledge of whether this is a graduated or an ‘all-or-nothing’ response to oxygen concentration would provide helpful information to better understand the molecular mechanisms controlling arrest and the resumption of development.

An impediment to quantifying embryonic development in oviparous animals is that the embryos of many species cannot be directly observed in a non-destructive manner. However, soon after the eggs of turtles and crocodilians are oviposited, they develop an opaque white spot on their upper surface, a process referred to as ‘chalking’ [1,28,29]. The white spot develops above the location where water is transferred from the albumen and eggshell into the yolk and sub-embryonic fluid. At this location, the vitelline membrane surrounding the embryo, sub-embryonic fluid and yolk merges with the shell membrane, causing localized dehydration of the eggshell, increasing its conductance and facilitating gas exchange with the embryo via the chorioallantois [30–32]. The chorioallantois is analogous to the lungs of the developing embryo and grows in step with the oxygen requirements of the embryo. As the embryo grows and its oxygen requirements increase, the white spot gradually increases in size to cover the entire eggshell. Since white spots do not appear on unfertilized eggs and rarely appear on eggs in which embryos have died before oviposition or very early in development [1,30], the appearance and subsequent growth of a white spot on the exterior of reptile eggs is generally accepted as a proxy for embryonic growth and development [12,25,26,30,32–34]. In addition, white spot growth and embryonic development are linked [5,6,32], and thus white spot formation is unlikely to be decoupled from embryonic development by the absence of oxygen since development does not proceed in anoxic conditions.

For this study, we propose that *C. mydas* embryos respond to hypoxic incubation in one of two ways. The first possibility is that there exists a critical ambient oxygen concentration, somewhere between 1% and 10% at sea level (approx. 8–76 mmHg) [35,36], below which *C. mydas* embryos are maintained in an arrested state, but above which arrest is broken and development proceeds normally and in a manner not dependent upon the actual oxygen concentration. The second possibility is that increasing hypoxia progressively slows development but that there is no critical oxygen concentration below which development is completely arrested, other than complete anoxia. To determine which of these scenarios is true, we determined the time of first appearance and rate of growth of the white spot during incubation of freshly oviposited eggs at various ambient oxygen concentrations, ranging from 1% to 9%.

## 2. Material and methods

### 2.1. Sample size calculation

The measured variables were time to white spot detection and rate of increase in white spot size. Sample sizes were calculated using the formula described by Noordzij *et al*. [37] and are based on values reported by Williamson *et al*. [26]. In that study, green turtle eggs incubated in normoxia at 28°C formed white spots in 39 ± 6.1 (mean ± s.d.) hours and the white spots increased in size by approximately 0.42% ± 0.17% (mean ± s.d.) of the total egg surface area per hour. At a 5% significance level, each treatment group required 30 eggs to have an 80% chance to detect an 11.5% increase in time to white spot formation between control eggs and eggs incubated in hypoxia. To detect a similar difference in growth rate would have required nearly 200 eggs in each treatment, a number we considered unjustifiably large. However, using 30 eggs in each group allowed us to detect a 30% difference in white spot growth rate between eggs in normoxic and hypoxic treatments, which we considered a reasonable trade-off between effect size and the number of eggs collected. Our actual sample sizes were 32 to 33 eggs per treatment group. This was approximately 10% larger than our power calculations and accounts for possible error or technical failures.

### 2.2. Egg collection

We collected 36 eggs from each of four adult female *C. mydas* and 25 eggs from each of two females (*n* = 194 eggs) on Heron Island, Queensland, Australia (23.44° S, 151.92° E) between 23 February and 1 March 2021. Eggs were collected by gloved hand as they were laid and placed on a bed of moist sand in a 21 l bucket. Immediately after collection, eggs were carried by hand to the laboratory, up to 650 m distant. All eggs were then cleaned with a soft brush to remove excess sand and individually labelled with a soft (6B) pencil before being placed into incubation chambers within 390 min (mean 223 min) of being laid.

### 2.3. Experimental design

All eggs were incubated in a temperature-controlled laboratory at the Heron Island Research Station. The mean temperature of the incubation room was 29°C and the temperature always remained between 27°C and 31°C except for one 4 h period when, due to power fluctuations, the temperature fell to 25°C before returning to 29°C.

Eggs from each clutch were randomly divided among six treatments with different ambient oxygen concentrations (less than or equal to 1%, 3%, 5%, 7%, 9% and 21%; table 1). Maximum (21%) and minimum (less than or equal to 1%) oxygen concentration treatments were applied to act as positive and negative controls, respectively, for embryonic development [19]. Eggs were incubated on a bed of moist beach sand (10% water v/v) in airtight Perspex incubators (Resi-Plex Plastics, Victoria, Australia) fitted with inflow and outflow ports. At the start of treatment and twice daily during treatment (mean interval 12.0 h, range 8.5–15.3 h) a mixture of air and pure (99.99%) nitrogen gas (BOC, Gladstone, Queensland) was pumped into the incubators until the desired oxygen level was reached (electronic supplementary material, figure S1). We monitored the gas mixture leaving the incubators by placing an oxygen sensor (Maxtec, Salt Lake City, Utah) connected to a data collector (Pasco, Roseville, California) in an outflow chamber. Oxygen concentration was sampled once per second and measured in parts per million, which was then converted into percentage of oxygen. Once the desired oxygen level was reached, the outflow chamber was disconnected and the inflow and outflow ports were sealed with aluminium foil affixed with surgical strapping tape (Leukoplast Sleek, BSN Medical, France). Total time in treatment averaged 72.1 h (range 71.1–74.1 h). This period was chosen because it is equal to the maximum time that sea turtle eggs may be chilled or stored in hypoxia without significantly increased embryonic mortality [12,17,38,39]. At the end of the treatment period, all eggs were carefully removed from the incubation chambers and placed in hand-dug nest cavities to a depth of 60 cm, mimicking the natural shape of green turtle egg chambers, and left undisturbed for the remainder of their incubation.

**Table 1.** Latency to detection and rate of growth of white spots on green turtle (*Chelonia mydas*) eggs when incubated at 29°C in various concentrations of oxygen for 72 h. Different superscript letters indicate statistically different groups. Treatments with the same letter were not significantly different according to the *post* -*hoc* Tukey HSD/Kramer test. SA_e_ = total calculated surface area of the egg. Eggs that showed white spots (WS) are said to be ‘chalked’. WS_prop_ = proportion of egg total surface area occupied by the white spot.

oxygen concentration (*n*)		1% (32)	3% (33)	5% (32)	7% (33)	9% (32)	21% (32)	*p*-values
no. chalked in treatment (%)		0 (0%)	15 (45%)	27 (84%)	33 (100%)	31 (97%)	32 (100%)	<0.001 (log-rank test)
hours to WS detection	min	n/a	49.5	27.4	23.6	23.7	19.1	
	max	n/a	72.1	74.0	71.9	44.6	42.0	
	mean (s.e.)	n/a	66.9^a^ (1.2)	51.6^b^ (2.8)	45.1^b^ (2.1)	33.7^c^ (1.1)	26.9^c^ (0.9)	<0.001 (ANOVA)
mean WS growth rate, WS_prop_ per hour (s.e.)		n/a	0.11^a,b,c^ (0.048)	0.10^a^ (0.012)	0.14^b^ (0.010)	0.23^c^ (0.009)	0.36^d^ (0.006)	<0.001 (ANCOVA)

On three occasions it was necessary to open an incubation chamber during treatment. On two occasions this was done so that eggs could be added to or removed from treatment, due to the limited number of chambers available. The third occasion was necessary to remove interior condensation so that unobstructed photographs of the white spots could be captured. Whenever a chamber was opened, eggs in hypoxic treatments were briefly exposed to the normoxic conditions in the laboratory. Five clutches were subjected to one such exposure event each, affecting 158 eggs. To reduce the possible impact on the embryos, pure nitrogen gas was pumped at 2 l m^−1^ for two minutes before the chamber was unsealed and during the time the chamber was open. Exposure time never exceeded 105 s and target % oxygen was restored within 5.3 min (electronic supplementary material, figure S2).

To monitor the appearance and growth of opaque white spots on the eggs, we took digital photographs (Samsung SM-G950U camera, 1950 **×** 4032 pixels, jpeg format) of each egg, from directly above, commencing from the start of treatment. Images were captured at the time the gas mixtures were refreshed and whenever new eggs were added to or removed from an incubation chamber, approximately every 12 h. Time between photographs averaged 12.6 h, although the timing of collection of photographic data varied due to logistical constraints imposed by egg collection efforts. Nevertheless, 91% of individual images were captured within 18 h of the previous photograph. At each photographic event, eggs were examined for the presence or absence of an opaque white spot. The proportion of eggs that developed white spots in each treatment was determined by dividing the number of eggs that developed white spots before the end of treatment by the total number of eggs in the treatment.

**Figure 1.**
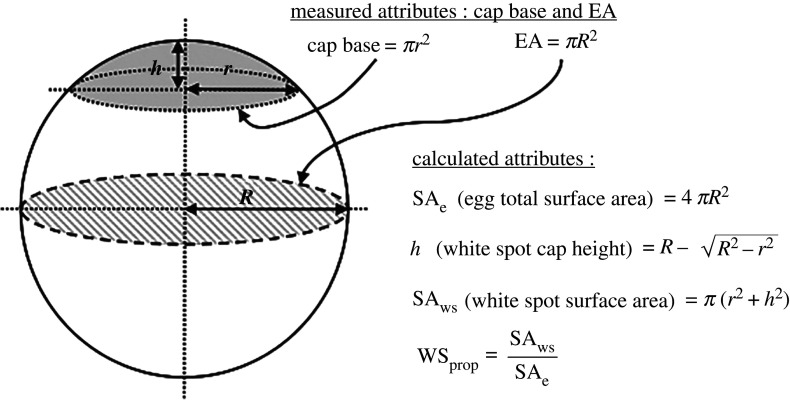
Mathematical basis for determination of egg surface area and white spot surface area. The diagonally shaded area indicates the plane used to mathematically define *R* (egg radius). *h* = height of spherical cap defined by white spot; *r* = radius of base of spherical cap defined by white spot (solid shading).

The size of *C. mydas* eggs varies within clutches laid by a single female and also among clutches laid by different females [40] and this size difference may confound direct comparisons of absolute white spot size. To avoid this confounding, we determined white spot size as a proportion of the total surface area of the egg (figure 1, details below). This method also avoids errors from variations in photographic distances and/or perspectives relative to the eggs. For most images at least two measurements were made of each egg and white spot, so mean values for each egg at each time point were determined.

The size of white spots was quantified using ImageJ image processing software [41]. Images were converted to tagged image file format (.tiff) and all measurements were performed on a monitor with a resolution of no less than 1080 **×** 1920 pixels measuring at least 35 cm (diagonal). For our analysis, eggs were assumed to be spherical [40,42] and white spots were treated as spherical caps. We calculated the total surface area of each egg (SA_e_) using the formula SA_e_ = 4*πR*^2^, where *R* = egg radius. To define *R*, we measured the area of each egg (EA), in pixels, in each image. EA was assumed to be equal to the area of a circle drawn by a plane intersecting the centre of the egg at its greatest visible diameter. EA was thus equal to *πR*^2^ and was multiplied by 4 to determine SA_e_. We then measured the area of each white spot as it appeared in the photo. The area thus captured was assumed to be equal to the area of the base of the spherical cap represented by the white spot, or *πr*^2^, where *r* = radius of white spot cap base. White spot surface area (SA_ws_) was determined using the formula SA_ws_ = *π* (*r*^2^ + *h*^2^), where h = white spot cap height, equal to *h* = *R* − √(*R*^2^ − *r*^2^). The proportion of each egg's total surface area occupied by the white spot (WS_prop_) was then calculated using the formula WS_prop_ = SA_ws_/SA_e_ (figure 1). The rate of growth of white spots was determined by comparing WS_prop_ in photos taken at successive time intervals.

### 2.4. Statistical analysis

All analyses were performed using R software [43]. Most data did not violate the assumptions of normality and homoscedasticity as assessed using the Plot package in RStudio software [44], so are presented as mean ± standard error. Two-tailed *p* ≤ 0.05 was considered statistically significant. Between-treatment differences in the proportions of eggs forming a white spot during the treatment period were assessed using the log-rank test for Kaplan–Meier survival curves. Between-group differences in the time to white spot detection (i.e. the estimate of the time to breaking of arrest) were assessed by one-way analysis of variance (ANOVA) followed by specific contrasts using the Tukey honestly significant difference (HSD) test, to which we applied Kramer's modification to account for unequal sample size. To assess between-group differences in the rate of growth of white spots, we created a linear mixed effects (LME) model using the lme4 package [45,46]. The LME model was constructed using time and oxygen concentration as fixed effects and, to control for maternal effects, maternal identity as a random effect. Analysis of covariance (ANCOVA), with independent variables treatment (categorical) and time (continuous), was then used to identify significant differences in mean growth rate between treatments.

## 3. Results

### 3.1. White spot development

White spots were detected (i.e. eggs **‘**chalked’) on 138 of the 194 eggs before the end of treatment (table 1). All eggs incubated in 21% oxygen formed a white spot but no eggs incubated at ≤1% oxygen did. The proportion of eggs forming a white spot during the treatment period varied with oxygen concentration (log-rank test, *χ*^2^ = 127, d.f. = 4, *p* < 0.001), being 45% at 3% oxygen, 84% at 5% oxygen, 100% at 7% oxygen, 97% at 9% oxygen and 100% at 21% oxygen. Mean time to white spot detection was progressively greater at lesser concentrations of oxygen (ANOVA, *F*_4,133_ = 52.64, *p* < 0.001). Between-group differences in time to white spot detection were statistically significant for all pair-wise comparisons except for 21% versus 9% oxygen (Tukey-Kramer, *p* = 0.06) and 7% versus 5% oxygen (*p* = 0.11).

### 3.2. White spot growth rate

**Figure 2.**
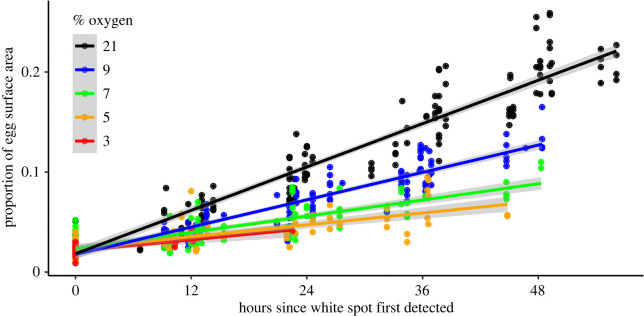
Growth of white spots on green turtle (*Chelonia mydas*) eggs during incubation at various ambient concentrations of oxygen. Symbols represent the proportion of the egg surface area covered by the white spot for individual eggs at various time points after the first appearance of the white spot. Solid lines are least-squares regression lines calculated from the linear mixed effect (LME) model. Shaded areas represent 95% confidence intervals of predicted values.

The rate of white spot growth was quasi-linear in all treatment groups and varied with oxygen concentration (ANCOVA, *F*_4,473_ = 149.5, *p* < 0.001) after controlling for maternal identity (figure 2). Mean white spot growth rate in all hypoxic treatments was significantly less than that in the positive control (21% oxygen) (Tukey-Kramer, *p* < 0.001). However, no significant difference was found between 3% oxygen and all other hypoxic treatments (table 1).

## 4. Discussion

Our results indicate that hypoxic incubation conditions delay the breaking of pre-ovipositional embryonic arrest and reduce the rate of embryonic growth in green sea turtles. We found no support for the hypothesis that a critical oxygen level exists, below which no visible development occurs. Rather, our observations support the alternative hypothesis of a direct relationship between oxygen concentration and development. The large proportion of eggs incubated in hypoxia in which we observed signs of development (82% of all eggs in 3–9% oxygen) during the 72 h treatment period suggests that green turtle embryos can survive moderately hypoxic conditions in the first days after oviposition, although rate of development is slowed.

No embryonic development was detected in eggs incubated in anoxia (less than or equal to 1% oxygen), confirming the proposition that pre-ovipositional arrest in turtles can be extended by anoxic incubation [12,17,19,25]. However, our results indicate that some green turtle embryos are able to break from pre-ovipositional arrest even when incubated at an oxygen concentration as low as 3%. During the 72 h treatment in hypoxia, white spots formed on 45% of eggs in the 3% oxygen treatment, suggesting that the embryos within these eggs were developing [1,30]. However, the latency between oviposition and appearance of the white spot was progressively extended by graded hypoxia, providing further evidence that embryonic development is slowed under hypoxic conditions [47]. The discovery in the current study that 3% oxygen is sufficient for turtle embryos to break arrest and resume development has important implications for research projects that use hypoxia as a method for safe transportation of turtle eggs [25]. Such projects should be robustly designed so that oxygen availability is kept at or below 1% at all times during translocation. For example, eggs should be placed in an incubator containing pure nitrogen immediately after oviposition and the level of ambient oxygen should be closely monitored until the eggs are placed in the hatchery nests.

The conclusion that embryonic development is progressively slowed by hypoxia is further supported by our observations regarding the rate of white spot growth in the various treatments. The size of the white spot is generally regarded as a proxy measure of embryonic growth [12,25,26,30,32–34]. The rate of growth of the white spot, and so by inference the development of the embryo, was progressively slower at lesser concentrations of oxygen. Interestingly, we observed a direct relationship between the rate of white spot growth and the concentration of oxygen. When considering both the time to white spot detection and the rate of white spot growth, the concentration of oxygen had a significant impact on how rapidly an embryo developed. Significant differences in time to white spot formation divided the treatments into four groups—anoxia, 3%, 5–7% and 9–21%—with each group breaking arrest earlier than those incubated at a lower oxygen concentration.

Our results indicate that moderate hypoxia in natural nests has the potential to influence early development of green sea turtle embryos. The lowest level of oxygen previously recorded in natural nests is approximately 10% [35,36], similar to the 9% oxygen treatment group in our current study. We did not detect significant differences in the proportion of eggs forming white spots or the time to formation of the white spot between the 9% and 21% treatment groups. However, there was a clear reduction in the rate of growth of the white spot between these two treatment groups. Our observations raise the possibility that longer incubation periods might result when new nests are laid in close proximity to older nests with more mature embryos which consume more of the available oxygen [36]. Hypoxia-induced prolongation of incubation may affect hatching success, particularly as temperature is expected to rise at many sea turtle rookeries due to global climate change [48]. Hatchlings exposed to high sand temperature during emergence from the nest may experience increased mortality due to heat stress [49–52]. Further, longer incubation duration increases the risk of negative impacts resulting from natural forces, such as tidal inundation/storms, predation and changing seasons (i.e. late-season nests in which the temperature cools to below the thermal tolerance range of embryos).

Oxygen-sensing pathways dependent on hypoxia-inducible factors have been identified as potentially important mediators of hypoxia-induced embryonic arrest in zebra fish [53] and green sea turtles [27]. Our current findings indicate that these mechanisms do not operate as a simple **‘**switch’, either permitting or blocking embryonic development. Rather, they appear to be progressively modulated by oxygen availability, so that embryonic development is directly dependent on the ambient oxygen concentration. This conclusion agrees with Gárriz *et al*.'s [27] report of differences in transcriptional activity between fresh-laid embryos, embryos incubated in normoxia and embryos in hypoxia-extended arrest. They found that embryos in all three groups were transcriptionally distinct. In combination, the results of our study and Gárriz *et al*. provide evidence that embryos in a state of hypoxia-extended arrest are not wholly **‘**static’ and that development proceeds on a molecular level, but along a different pathway when compared with normoxic incubation. Further, their finding that laboratory hypoxic incubation has the potential to increase the activity of some hypoxia-inducible factors while decreasing the activity of others suggests that the oviducal environment somehow maintains oxygen homeostasis in a way that is interrupted when eggs are incubated in nitrogen. This highlights the marked difference between maintaining eggs within a fluid medium and the hypoxic incubation techniques used to date. We hypothesize the existence of a signalling pathway which may maintain the stability of pre-ovipositional embryonic arrest. An investigation aimed at determining the presence or absence of signalling molecules in oviducal fluids is warranted.

One of the limitations of this study is the relatively small sample size. Thus, we cannot exclude the possibility that the lack of a statistically significant difference between the growth rate of the 3% treatment and that of the 5%, 7% and 9% treatments may have been a type 2 error attributable to the fact that only 15 eggs (45% of that treatment group) showed evidence of chalking in the 3% treatment. Nevertheless, the mean growth rate (i.e. the increase in the proportion of egg surface area per hour) in the 9% oxygen treatment was more than double that of the 3% treatment. Thus, taken collectively, our data provide compelling evidence that increasing hypoxia progressively slows early development of green sea turtle embryos.

We acknowledge that our indirect observations (of white spot growth) require confirmation with direct observations of embryonic development. Although unlikely, it is possible that hypoxic incubation disrupts physiological processes and decouples white spot formation and subsequent growth from embryonic development. It should be noted, however, that the white spot is essentially a part of the developing respiratory organ of the embryo, the chorioallantois. As such, its formation and increase in size occurs contemporaneously with the development of other embryonic organ systems. It remains to be demonstrated that sea turtle ontogeny is altered by hypoxic incubation. If our hypoxic treatments had decoupled the connection between embryonic growth and white spot development, it is unlikely that the behaviour of the white spots would have been as consistent and repeatable as we observed. Although beyond the scope of the present study, there is a need for experimental validation of the pervasive, assumed correlation between white spot size and embryonic developmental stage.

## 5. Conclusion

Our findings indicate that the rate of early embryonic development in green turtles decreases with decreasing oxygen concentration. We were unable to identify a critical ambient oxygen concentration below which arrest is maintained, other than almost complete anoxia (less than or equal to 1% oxygen). We found evidence that the moderately hypoxic conditions probably found in newly laid nests which are located near very mature nests (i.e. at or near 10% oxygen) can significantly reduce the rate of embryonic development when compared with conditions found in more spatially distant nests. Thus, some nests at high-density nesting beaches may have extended incubation periods which, when combined with increasing temperature, can increase hatchling mortality. However, we found evidence that green turtle embryos are resilient to hypoxic conditions and are capable of development in moderate to severe hypoxia, albeit at a reduced rate. Additional research is necessary to better understand the impact of moderate hypoxia on later stages of embryonic growth when experienced by early-stage embryos.

## Data Availability

The datasets supporting this article have been uploaded as part of the Supplementary Material [54]. Electronic supplementary material is available online at [55].
